# Primary stability of different implant macrodesigns in a sinus floor elevation simulated model: an ex vivo study

**DOI:** 10.1186/s12903-022-02345-5

**Published:** 2022-08-08

**Authors:** Mikio Imai, Yoichiro Ogino, Hideaki Tanaka, Kiyoshi Koyano, Yasunori Ayukawa, Takeshi Toyoshima

**Affiliations:** 1 Section of Implant and Rehabilitative Dentistry, Division of Oral Rehabilitation, Faculty of Dental Science, Kyushu University, 3-1-1 Maidashi, Higashi-ku, Fukuoka, 812-8582 Japan; 2 Section of Fixed Prosthodontics, Division of Oral Rehabilitation, Faculty of Dental Science, Kyushu University, Fukuoka, Japan; 3 Tanaka Dental Clinic, Itoshima, Fukuoka, Japan; 4 Division of Advanced Dental Devices and Therapeutics, Faculty of Dental Science, Kyushu University, Fukuoka, Japan; 5 Toyoshima Dental Clinic, Takamatsu, Kagawa Japan

**Keywords:** Dental implant, Primary stability, Sinus floor elevation, Ex vivo model, Maximum insertion torque, Implant stability quotient

## Abstract

**Background:**

A novel type of implant (Straumann® BLX implant) has been developed for certain stability from the mechanical and biological aspects and is expected for the implant placement in atrophic maxilla with sinus floor elevation (SFE).

**Purpose:**

The aim of this study was to evaluate the primary stability in the implants with different macrodesigns in an SFE simulated model. Primary stabilities defined as maximum insertion torque (MIT) and implant stability quotient (ISQ) were compared between this novel type of implant and other types.

**Materials and Methods:**

Five types of Straumann® 10 mm length implants (Standard Plus; SP, Tapered Effect; TE, Bone Level; BL, Bone Level Tapered; BLT and BLX) and two types of Straumann® 6 mm length implants (SP short, BLX short) were used in this study. Each implant was inserted through 5 mm–thick porcine iliac crest blocks (an SFE simulated model). Primary stability was evaluated by using MIT and ISQ.

**Results:**

The mean value of MIT for BLX group showed significantly higher values than SP, BL (*p* < 0.01), and TE (*p* < 0.05) groups. The mean value of ISQ for BLX group was significantly higher than the other groups (*p* < 0.01). The mean value of MIT and ISQ for BLX and BLX short group were significantly higher than those for SP and SP short group (*p* < 0.01).

**Conclusions:**

In an SFE simulated ex vivo model, BLX group showed the highest values. These results suggest that implant selection can play a crucial role in the achievement of primary stability during SFE and simultaneous implant placement.

**Supplementary Information:**

The online version contains supplementary material available at 10.1186/s12903-022-02345-5.

## Introduction

Sinus floor elevation (SFE) has been known as a surgical technique to increase the vertical height for implant placement in the atrophic maxilla [1, 2]. Two surgical procedures for SFE have been known: the lateral window technique and the crestal osteotome technique [1]. The previous studies reported that both techniques were clinically reliable due to high success rates of implants after SFE [3–6]. However, there are some considerations to decide surgical procedures such as the preexisting bone height between sinus floor and maxillary bone crest, and the timing of implant placement [2, 7]. In addition, a recent review discussed the necessity of graft materials in SFE [7] and several studies and reviews reported that SFE with or without graft materials was clinically acceptable based on vertical bone gain, peri-implant marginal bone loss and implant survival [8–10]. The created space between the elevated Schneiderian membrane and the osseous floor of the maxillary sinus should be maintained by graft materials or a simultaneously placed implant like a tent pole (tenting technique) [11]. In SFE without graft materials, primary stability must be achieved by preexisting maxillary bone.

Primary stability is defined as the mechanical stability upon implant insertion, resulting from initial contact of the implant with the bony wall of the osteotomy [12]. It has been well known that primary stability also plays a crucial role in the achievement of osseointegration [12]. Primary stability is affected by multiple factors, including bone density, surgical technique and implant design [12–18]. Above all, implant design selection can be a predictable factor for higher primary stability. Although various implant designs have shown greater stability in dense bone, primary stability can decrease remarkably in low-density bone [13]. Suitable implant designs should be considered to achieve primary stability in low-density bone. In particular, the positive effects of macrodesigns of implant such as implant shape, thread shape, thread pitch, depth, thickness and face angle on primary stability have been suggested [19–22]. Multiple designs of implants have been developed and are commercially available. As one of these implants, a novel tapered implant with a distinctive threaded design (Straumann® BLX implant) is currently available on the market. This implant system has been developed for certain stability from the mechanical and biological aspects and is expected for the implant placement in atrophic maxilla with SFE. To ensure the availability, the effect of this type of implant on primary stability and the comparisons with other types would be required.

Therefore, the aim of this study was to evaluate the primary stability in the implants with different macrodesigns in an SFE simulated model. Primary stabilities defined as maximum insertion torque (MIT) value and implant stability quotient (ISQ) value were compared between this novel type of implant and other types from the same company. In addition, the effects of the length of implants on primary stability were also evaluated.

## Materials and methods

### Specimens for an SFE simulated model (an ex vivo model)

**Fig. 1:**
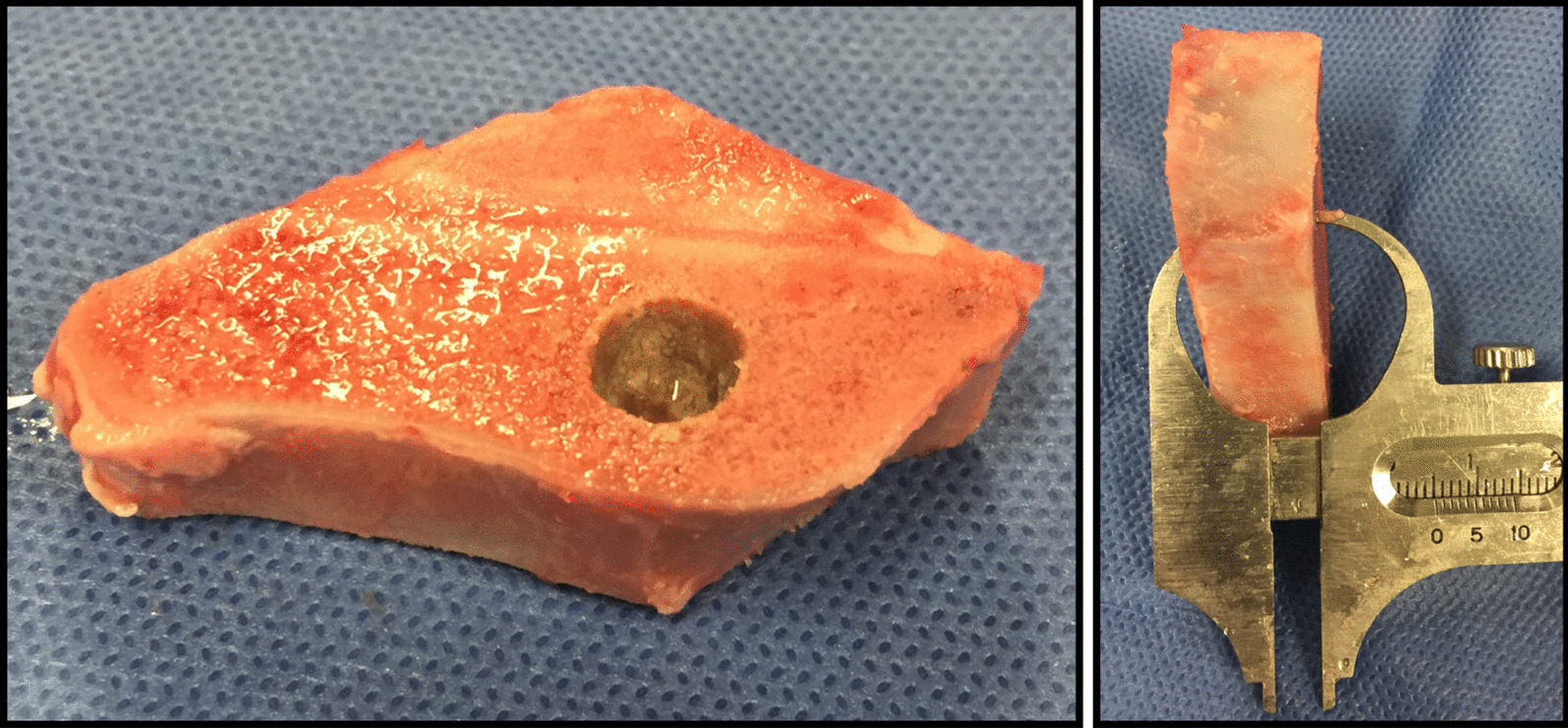
Bone blocks created from porcine iliac crest (approximately 50 mm × 20 mm × 5 mm). Fourty-two fresh blocks were then randomly chosen for this study. After removal of the adjacent soft tissue, the surfaces of the bone blocks were thoroughly cleaned by rinsing in water. Each block was checked macroscopically for irregularities, and the thickness of 5 mm was verified using a precision caliper

Porcine iliac crest was used to create bone blocks (approximately 50 mm × 20 mm × 5 mm) using a water-cooled precision diamond saw (YSC-500FDX, Yutaka, Aichi, Japan) and round bur (Fig. 1). Six blocks were prepared from one specimen and totally, 42 fresh blocks were used for this study. After removal of the adjacent soft tissue, the surfaces of the bone blocks were thoroughly cleaned by rinsing in water. Each block was checked macroscopically for irregularities, and the thickness of 5 mm was verified using a precision caliper. This thickness was designed to simulate the preexisting atrophic maxillary bone.

### Implants

**Fig. 2:**
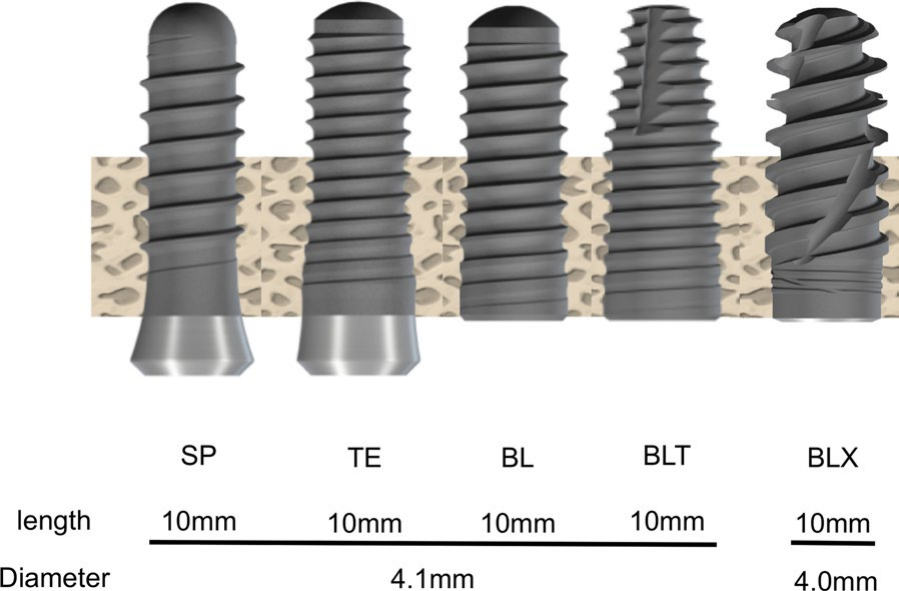
Schematic images of five Straumann implants. SP, Standard Plus implant 10 mm; TE, Tapered Effect implant 10 mm; BL, Bone Level implant 10 mm; BL, Bone Level Tapered implant 10 mm; BLX, BLX implant 10 mm

**Fig. 3:**
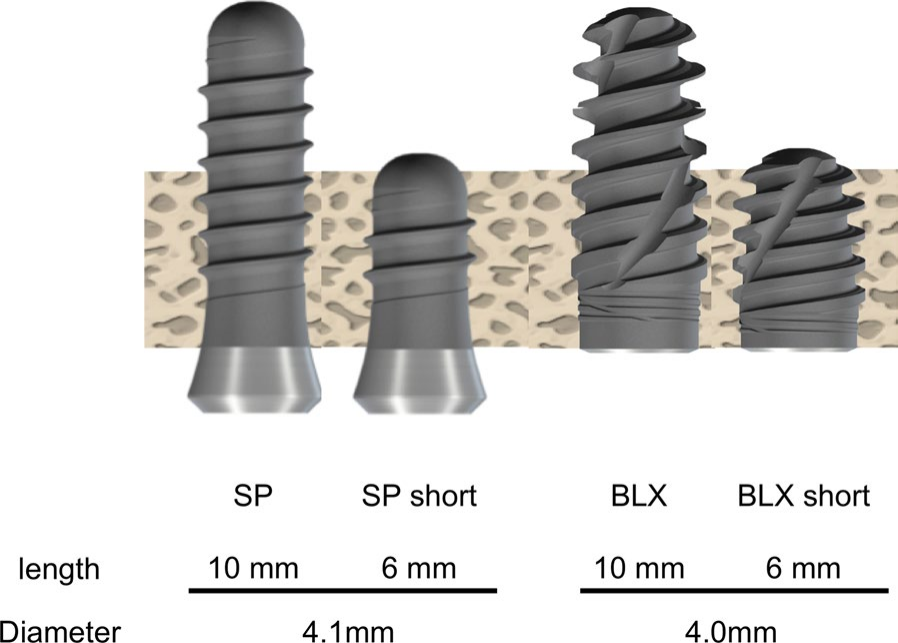
Schematic images of four Straumann implants. SP, Standard Plus implant 10 mm; SP short, Standard Plus implant 6 mm; BLX, BLX implant 10 mm; BLX short, BLX implant 6 mm

Implants used in this study were shown in Tables 1 and 2. (Figs. 2 and 3).

**Table 1:** Five kinds of implants to evaluate the effect of macrodesigns on primary stability

	Implant	Drilling size
①SP	Straumann® Standard Plus implants (SP; length 10 mm, diameter 4.1 mm)	Φ 3.5
②TE	Straumann® Tapered Effect implants (TE; length 10 mm, diameter 4.1 mm)	Φ 3.5
③BL	Straumann® Bone Level implants (BL; length 10 mm, diameter 4.1 mm)	Φ 3.5
④BLT	Straumann® Bone Level Tapered implants (BLT; length 10 mm, diameter 4.1 mm)	Φ 3.5
⑤BLX	Straumann® BLX implant (BLX; length 10 mm, diameter 4.0 mm)	Φ 3.5

**Table 2:** Four kinds of implants to evaluate the effect of macrodesigns and length on primary stability

	Implant	Drilling size
①SP	Straumann® Standard Plus implants (SP; length 10 mm, diameter 4.1 mm)	Φ 3.5
②SP short	Straumann® Standard Plus short implants (SP short; length 6 mm, diameter 4.1 mm)	Φ 3.5
③BLX	Straumann® BLX implant (BLX; length 10 mm, diameter 4.0 mm)	Φ 3.5
④BLX short	Straumann® BLX short implant (BLX short; length 6 mm, diameter 4.0 mm)	Φ 3.5

All implants had SLActive surface. Thread pitch of each implant was as follows: SP = 1.25 mm, TE, BL and BLT = 0.8 mm, BLX = 1.125 mm and BLX short = 0.9 mm.

All implants and surgical kits were provided from Straumann (Straumann®, Institute Straumann AG, Basel, Switzerland).

#### Implant placement in an SFE simulated model

The implants were placed into the bone blocks with the thickness of 5 mm (an SFE simulated model). Two persons performed the implant placements. The order of placement was randomized.

These implants were divided into 2 groups: 1) Five implants with the length of 10 mm to evaluate the effect of macrodesigns on primary stability, 2) SP and BLX with the length of 10 mm and 6 mm to evaluate the effect of macrodesigns and the lengths on primary stability. All implants were inserted from the cutting cross sections, which were consisted of only cancellous bone, according to the conventional insertion protocol of the manufacturer (Straumann®, Institute Straumann AG, Basel, Switzerland). Drilling was allowed to penetrate completely through the blocks and final drill diameter was defined as 3.5 mm. The maximum insertion torque (MIT) values during implant insertion were recorded with the digital torque driver (CEDAR, Sugisaki Keiki, Ibaraki, Japan). Each group was consisted of 6 implants.

#### Implant stability quotient (ISQ) value

Bone resonance frequencies were measured by Osstell^TM^ Mentor resonance frequency analysis transducer (Model 6.0, Integration Diagnostics, Göteborg, Sweden). The transducers were mounted on the implants and tightened with a screw by hand. The frequency response of the system was recorded following manufacturer's instruction and the measurements were performed from four directions (from left, right, front and back).

### Statistics

Statistical analyses were performed using SPSS software (version 16.0, SPSS Inc., Chicago, IL, USA). Statistical significance of the differences between the groups was determined by Tukey test following one-way ANOVA. *p* Values of less than 0.05 were considered to be significant.

## Results

### The effects of macrodesigns on primary stability

#### MIT values

**Fig. 4:**
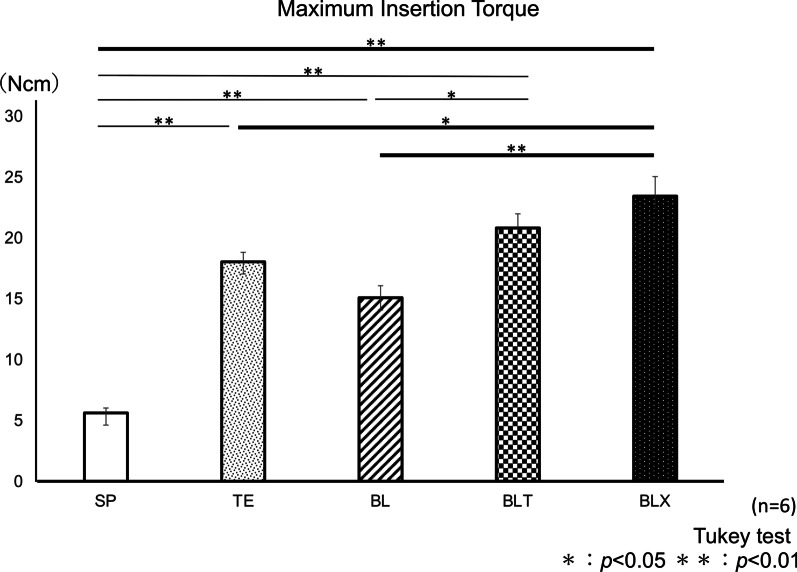
Mean values of maximum insertion torque of five implants with different  implant designs. *p < .05, **p < .01

The mean values of MIT in 10 mm length implants were shown in Fig. 4. BLX group showed significantly higher mean value than SP, BL (*p* < 0.01, respectively), and TE (*p* < 0.05) groups. BLT group showed significantly higher mean value than BL (*p* < 0.05) and SP (*p* < 0.01) groups. There were also significant differences between BL and SP, and TE and SP (*p* < 0.01, respectively).

#### ISQ values

**Fig. 5:**
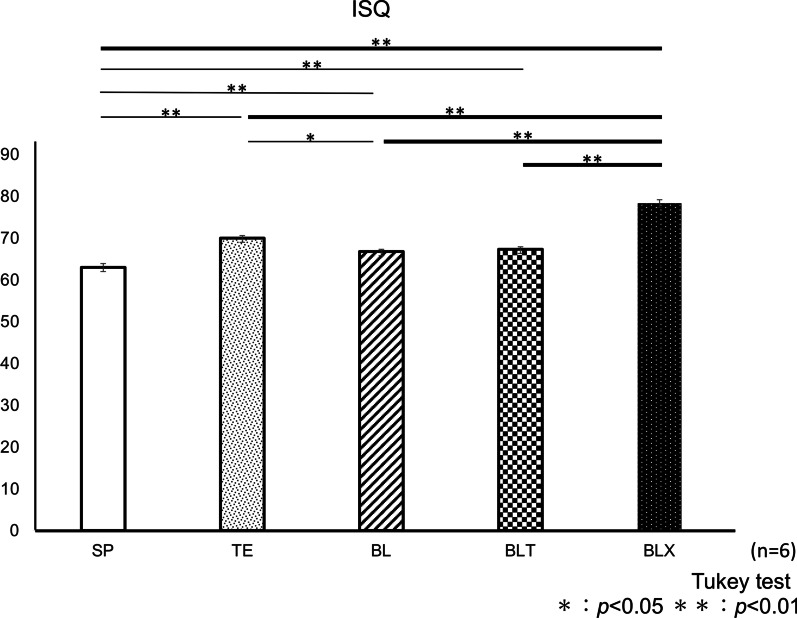
Mean values of implant stability quotient of five implants with  different  implant designs. *p < .05, **p < .01

The mean values of ISQ were shown in Fig. 5. The mean value of BLX group was significantly higher than the other groups (*p* < 0.01, respectively). The mean value of SP group was significantly lower than that of TE, BL and BLT group (*p* < 0.01, respectively) in addition to BLX group. The mean value of TE group was significantly higher than that of BL group (*p* < 0.05).

### The effects of macrodesigns and the lengths on primary stability

#### MIT values

**Fig. 6:**
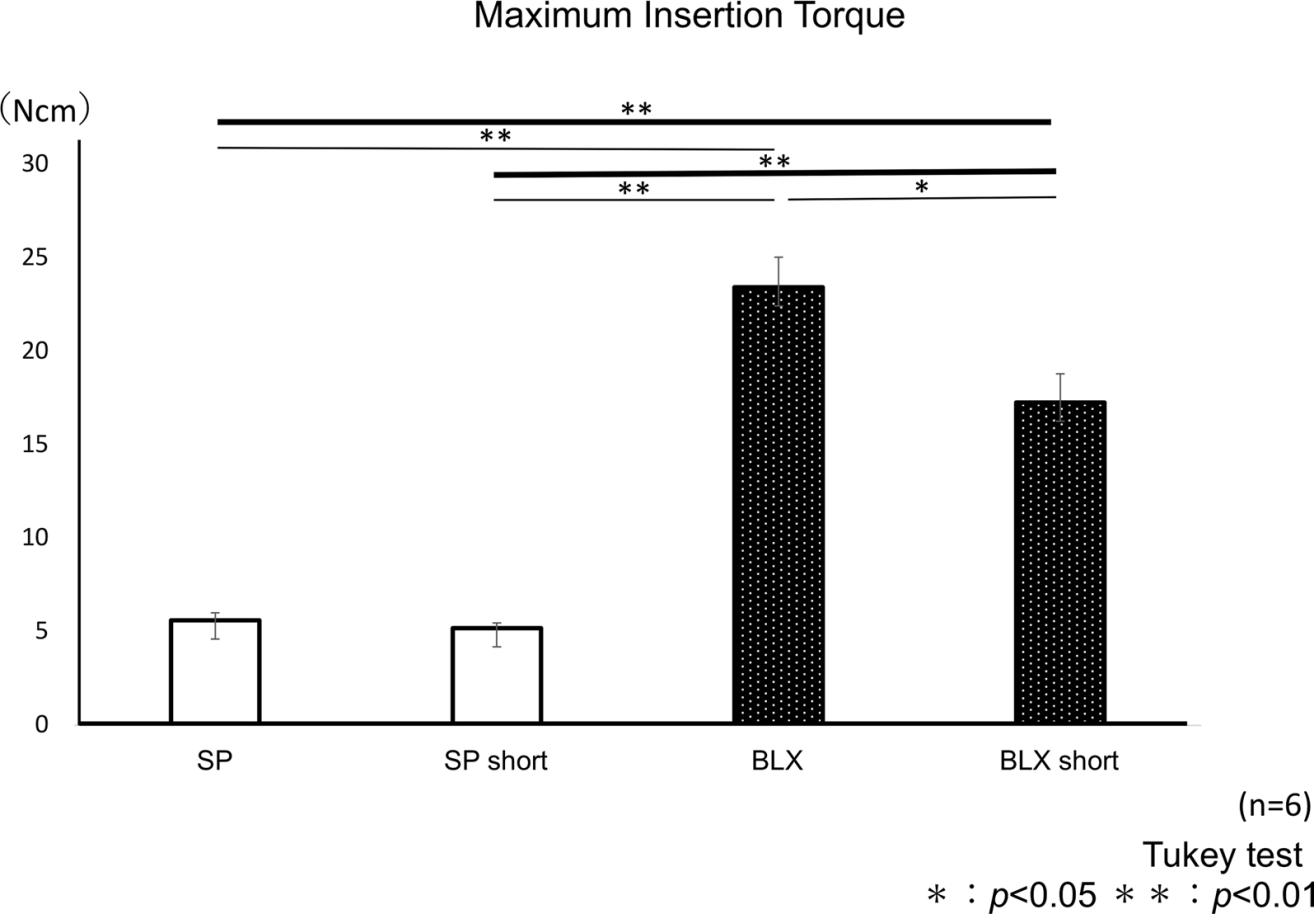
Mean values of maximum insertion torque of four implants with  different  implant lengths. *p < .05, **p < .01

The mean values of MIT were shown in Fig. 6. The values of BLX and BLX short group were significantly higher than that for SP and SP short group (*p* < 0.01, respectively). The value of BLX group was significantly higher than that of BLX short group (*p* < 0.05), but no difference was identified between SP and SP short groups.

#### ISQ values

**Fig. 7:**
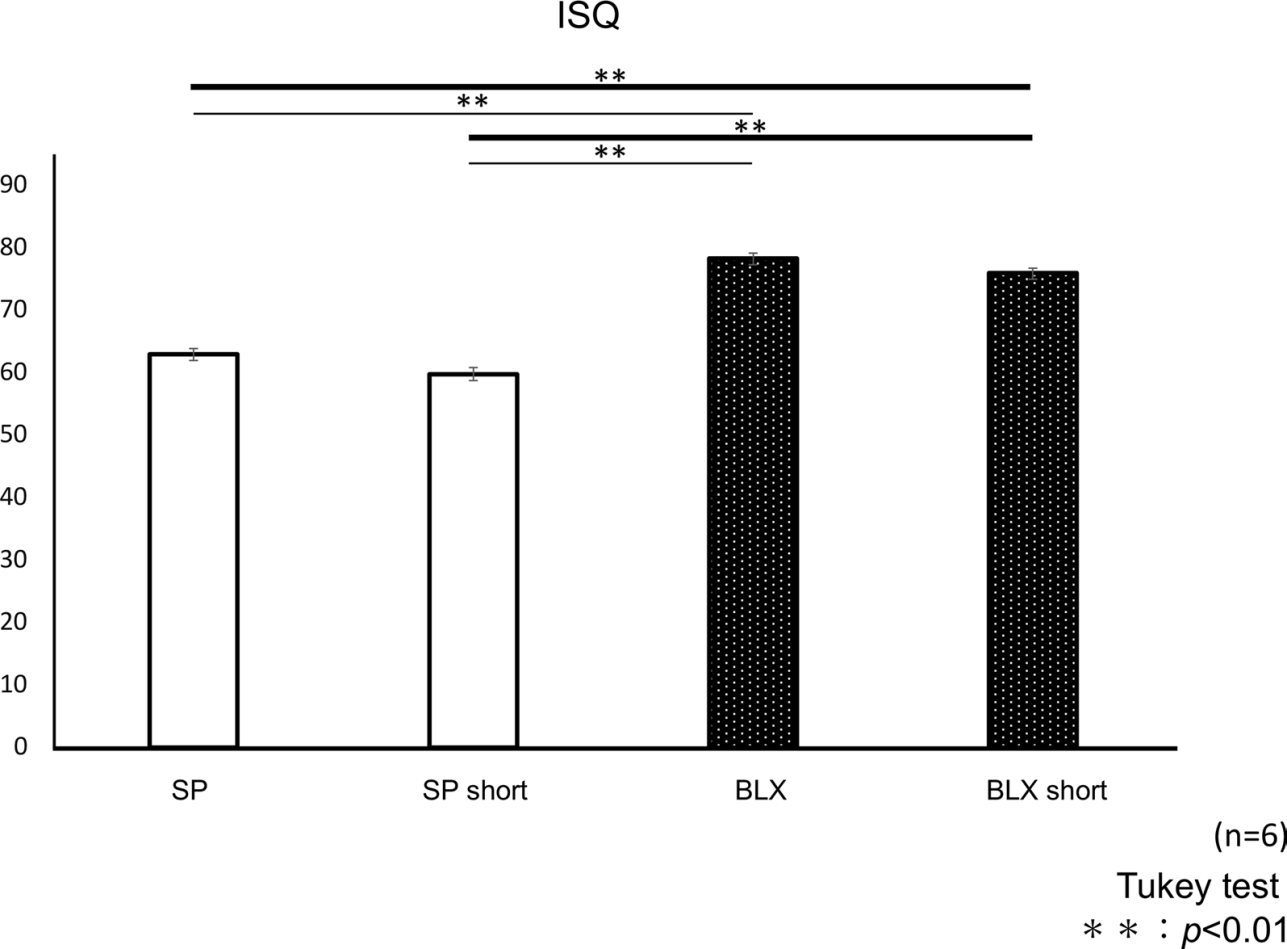
Mean values of implant stability quotient of four implants with different  implant lengths. **p < .01

The mean values of ISQ were shown in Fig. 7. The mean values of BLX and BLX short group were significantly higher than those of SP and SP short group (*p* < 0.01, respectively). However, there were no significant differences between 10 and 6 mm implants in the same macrodesigns.

## Discussion

Primary stability is attributed to bone quantity and quality, implant macrodesign and surgical technique [12–18]. Surgical procedure for implant placement in the edentulous posterior maxilla must be planned with caution due to limited anatomical structure and critical bone quality [1, 2]. Especially, SFE might be required to place the implants in the atrophic maxilla, and primary stability should be achieved with the preexisting bone during simultaneous implant placement [2]. Previous studies clearly demonstrated that the effect of implant macrodesign on primary stability [12–18]. Above all, thread pattern and implant body shape can contribute to primary stability and can be selected by the surgeons during preoperative planning. This study aimed to compare the primary stability depending on the macrodesigns between the novel type of implant and other types in an SFE simulated model. In addition, the effect of the lengths on primary stability was also evaluated.

At first, an ex vivo model was used to analyze primary stability. Bone blocks such as bovine rib, bovine kneecaps, porcine iliac crest and cow femur were used to evaluate primary stability in the previous studies[14–18, 21]. In this study, all implants were inserted from cross-sectional cancellous bone, not including cortical bone, which simulated poor maxillary bone. The thickness of 5 mm which was required for favorable results of SFE [2, 7], was designed to simulate the preexisting maxillary bone during SFE and all implants should penetrate completely through the blocks and this model was supposed to be valid. And six blocks were made from one specimen, so there should be no significant difference in bone density.

The present experiment assumes sinus floor elevation and simultaneous implant placement without bone graft. The residual bone height required for this is listed as 5 mm [7], 5.7 mm [9], 4.2 mm with bone graft, and 4.5 mm without bone graft [10].　If the residual bone height is 4 mm or less, staged implant placement after sinus floor elevation is recommended [2, 7].

BLX group with tapered design, comparatively wider thread pitches (1.125 mm) and larger thread depth and width, presented the highest MIT and ISQ values among the implants with the length of 10 mm. Regarding thread pitches, although the previous review noted that the implants with more threads could achieve a higher bone-implant contact and stronger resistance to vertical load [19], the effects of all factors of thread designs on primary stability have been still unclear. However, a previous report showed that thread pitches, depth and width were associated with primary stability [23]. Furthermore, a recent previous study showed that narrower threads could create higher primary stability [22]. This study showed the features of BLX which were designed to enhance primary stability could enhance MIT and ISQ values in an SFE simulated model, although the features of BLX group were not in agreement with some previous findings. In addition, BLT group showed significantly higher MIT than BL and SP groups, not TE group. BLT and TE groups had tapered designs and comparatively narrower threads pitches (0.8 mm), and these might play an important role in higher MIT and ISQ values. Although BL and SP groups had parallel designs, the former had more threads due to narrower thread pitches (BL vs SP: 0.8 vs 1.25 mm), resulting in significantly higher MIT and ISQ values. These results suggested that multiple factors were associated with primary stability (MIT and ISQ values), even in an SFE simulated model that was designed to evaluate primary stability with a limited bone volume. A recent previous study that compared the primary stability between BLX implants and TE implants in a similar model (sinus lift-simultaneous implant insertion model) indicated that TE implants with wider diameter showed higher ISQ values [24]. The difference between this previous study and the present study was bone condition for implant placement. The bone specimens in this previous study were consisted of cortical bone and cancellous bone, and it was suggested that TE implants could indicated higher ISQ values because TE implants with tapered neck design could achieve higher primary stability at cortical bone. An SFE simulated model in the present study was more severe situation, which was only cancellous bone, and it was supposed that BLT and BLX groups could enhance primary stability through other mechanisms.

The limited bone volume might allow the placement of only short length implant. This study also evaluated the effect of implant length on primary stability. This study clearly demonstrated that BLX groups could achieve significantly higher MIT and ISQ values than SP groups regardless of the length of implants. The effect of implant length on primary stability (ISQ values) was investigated in a previous study and it suggested higher ISQ values in longer implants [25]. However, available bone thickness was limited in the present study. Interestingly, BLX group demonstrated significantly higher MIT values than BLX short and both SP groups, although there were no differences ISQ values between BLX and BLX short groups. The differences between BLX group and BLX short group were not only the length but also the thread pitches (BLX: 1.125 mm, BLX short: 0.9 mm). Unfortunately, the detailed mechanisms of BLX macrodesigns for enhancing primary stability could not be elucidated. However, this simulated model clearly identified the effective features in the achievement of primary stability. One more unfortunate thing was that the effects of the length on primary stability were not investigated in all types of implants. This is because 6 mm short type implants are only available in SP and BLX. Further studies will be expected in a similar situation.

This study had several limitations. Though primary stabilities of five implant types were investigated, these results cannot directly be applied to further osseointegration (secondary stability) and load-bearing capacity after the healing period. Satisfactory clinical outcomes of implants after SFE with or without graft materials have been reported [8–11], although primary stability is one of the most important factors for osseointegration. Clinical investigations using several types of implants for SFE and simultaneous placement would be favorable to compare the macrodesigns and primary stabilities among these implants. In addition, these results could be applied to this ex vivo model (porcine iliac crest with the thickness of 5 mm), although the effects of macrodesign and length of implants on primary stability could be evaluated. The effects of other designs or other lengths should be evaluated in various thicknesses. In clinical situations, the most important factor is the primary stability, not the residual bone height, and the surgeons should pay attention to it during simultaneous placement with SFE [2, 7]. However, the results of this study suggested that implant macrodesign could contribute to the enhancement of the primary stability in the limited residual bone height.

## Conclusion

In an SFE simulated ex vivo model, the primary stabilies defined by MIT and ISQ values were affected by the implant macrodesigns. And the BLX short group was significantly higher than the SP and SP short groups.

Especially, BLX group showed the highest values and the other implants might be clinically acceptable based on the previous reports. These results suggest that implant selection can play a crucial role in the achievement of primary stability during SFE and simultaneous implant placement.

## Supplementary Information

**Additional file 1.** The datasets analyzed during the current study are available at https://doi.org/10.1186/s12903-022-02345-5.** Table 1**. MIT values of 5 kinds of implants.** Table 2**. MIT mean values.** Table 3**. ISQ values of 5 kinds of implants from 4 directions.** Table 4**. ISQ mean values.** Table 5**. MIT values of 4 kinds of implants. **Table 6**. MIT mean values.** Table 7**. ISQ values of 4 kinds of implants from 4 directions.  **Table 8**. ISQ mean values.

## Data Availability

The data that support the findings of this study are available from the first and corresponding authors upon reasonable request.
