# Supplementary material for: Primary stability of different implant macrodesigns in a sinus floor elevation simulated model: an ex vivo study
Source: BMC Oral Health. 2022 Aug 8;22:332. doi: 10.1186/s12903-022-02345-5 (PMC9361700; doi:10.1186/s12903-022-02345-5)
Supplement: Supplementary file 1 — Additional file 1. The datasets analyzed during the current study are available at https://doi.org/10.1186/s12903-022-02345-5. Table 1. MIT values of 5 kinds of implants. Table 2. MIT mean values. Table 3. ISQ values of 5 kinds of implants from 4 directions. Table 4. ISQ mean values. Table 5. MIT values of 4 kinds of implants. Table 6. MIT mean values. Table 7. ISQ values of 4 kinds of implants from 4 directions. Table 8. ISQ mean values. [file 12903_2022_2345_MOESM1_ESM.pdf]

Table 1 MIT values of 5 kinds of implants

SP	TE	BL	BLT	BLX
4.5	16.6	14	26.2	18
5.7	20.3	15	18.2	23.1
7.6	16.9	17.6	18.7	21.3
4.9	20.9	18.5	18.3	23.5
5.2	17.7	11.2	22	31.1
5.8	15.8	14.2	21.5	23.6

Table 2 MIT mean values

	SP	TE	BL	BLT	BLX
Average	5.61666667	18.0333333	15.0833333	20.8166667	23.4333333
SD	0.40511772	0.77805551	0.98669388	1.16079874	1.60721752

Table 3 ISQ values of 5 kinds of implants from 4 directions

SP	TE	BL	BLT	BLX
53	67	64	65	79
53	67	64	66	70
55	67	64	66	76
59	67	64	70	76
66	67	68	64	80
65	67	68	66	69
62	67	68	76	82
65	72	64	66	72
68	75	69	65	78
65	70	70	64	85
68	73	70	68	74
68	67	70	65	74
64	72	68	68	76
64	77	70	65	76
63	74	68	68	82
63	74	72	68	82
64	72	66	71	84
67	70	68	71	80
67	70	66	71	80
67	70	65	70	80
56	70	63	65	82
63	67	65	66	82
63	67	63	65	80
63	70	65	66	80

Table 4 ISQ mean values

	SP	TE	BL	BLT	BLX
Average	62.9583333	69.9583333	66.75	67.2916667	78.2916667
SD	0.90997399	0.6137294	0.52786914	0.58475769	0.8508149

Table 5 MIT values of 4 kinds of implants

SP	SP short	BLX	BLX short
4.5	5.3	18	12.7
5.7	5.9	23.1	15.5
7.6	6.1	21.3	23.4
4.9	4.1	23.5	18.4
5.2	5.1	31.1	13.5
5.8	4.7	23.6	20.1

Table 6 MIT mean values

	SP	SP short	BLX	BLX short
Average	5.61666667	5.2	23.4333333	17.2666667
SD	0.40511772	0.27788887	1.60721752	1.53743413

Table 7 ISQ values of 4 kinds of implants from 4 directions

SP	SP short	BLX	BLX short
53	58	79	73
53	58	70	74
55	61	76	70
59	57	76	70
66	59	80	76
65	59	69	76
62	59	82	80
65	59	72	80
68	70	78	71
65	66	85	75
68	61	74	80
68	66	74	80
64	62	76	80
64	67	76	80
63	67	82	75
63	66	82	75
64	53	84	80
67	56	80	80
67	60	80	80
67	60	80	80
56	56	82	70
63	49	82	74
63	56	80	70
63	49	80	74

Table 8 ISQ mean values

	SP	SP short	BLX	BLX short
Average	62.9583333	59.75	78.2916667	75.9583333
SD	0.90997399	1.07891728	0.8508149	0.78279939
